# A pilot study of blood supply of the coracoid process and the coracoid bone graft after Latarjet osteotomy

**DOI:** 10.1042/BSR20190929

**Published:** 2019-11-12

**Authors:** Zhenhan Deng, Daqiang Liang, Weimin Zhu, Haifeng Liu, Jian Xu, Liangquan Peng, Xuchun Li, Ying Li, Ronak Naveenchandra Kotian, Wei Lu, Daping Wang

**Affiliations:** 1 Department of Sports Medicine, The First Hospital Affiliated to Shenzhen University, Shenzhen Second People’s Hospital, Shenzhen, Guangdong, China; 2 Key Laboratory of Tissue Engineering of Shenzhen, The First Affiliated Hospital of Shenzhen University, Shenzhen Second People’s Hospital, Shenzhen, Guangdong, China; 3 Department of Orthopaedics, Victoria Hospital, Bangalore Medical College and Research Institute, Bangalore, India

**Keywords:** blood supply, conjoint tendon, coracoid process, Latarjet, micro-CT

## Abstract

Latarjet osteotomy is still one of the most reliable and commonly used surgeries in treating recurrent anterior shoulder dislocation. The coracoid process (CP) is the main structure of this surgery. However, the blood supply of CP is not fully understood, and the extent of destruction of blood supply of coracoid bone graft after Latarjet osteotomy procedure is still controversial. Five embalmed cadaveric upper limbs specimens were employed for macro observation of the blood supply of CP. The conjoint tendon (CT) and CP interface were dissected for histology. Sixteen fresh frozen shoulder specimens were used for perfusion and micro CT scanning. Eight specimens were used to present the whole vessel structure of CP. The other eight underwent Latarjet osteotomy procedure. The coracoid bone grafts in both groups were scanned to clarify the remnant blood supply. It was found that the CP was nourished by supra-scapular artery (SSA), thoracic-acromial artery and branch from second portion of the axillary artery (AA). After Latarjet osteotomy procedure, no artery from CT was detected to penetrate the CP at its attachment. Only in one specimen the blood vessel that originated from the CT penetrated the bone graft at the inferior side. Therefore, most of the blood supply was destroyed although there is a subtle possibility that the vessels derived from the CT nourished the inferior side of the CP. In a nutshell, CP is a structure with rich blood supply. The traditional Latarjet osteotomy procedure would inevitably cut off the blood supply of the coracoid bone graft.

## Introduction

Anterior shoulder dislocation is the most common type of joint dislocation. The process of dislocation can cause glenoid bone defects due to impingement between humeral head and antero-inferior glenoid rim [1].

Latarjet procedure is an effective method to repair the defective glenoid in the treatment of recurrent anterior dislocation of the shoulder joint [2,3]. The operation was first proposed by the French surgeon Latarjet in 1954 [4]. The mainstay of the procedure includes osteotomy of horizontal part of the coracoid process (CP), deattachment of the pectoralis minor (PM), the coracoacromial ligament (CAL) and the coracohumeral ligament (CHL) from the CP, leaving only the attachment of conjoint tendon (CT) on the CP. After that, the coracoid bone graft is transferred through a split of subscapularis (SUBS) to the defective glenoid and fixed. The transferred coracoid bone graft is expected to heal and prevent the humeral head from dislocating off the glenoid. CP is a structure with many origins and insertion of muscles and ligaments. Therefore CP osteotomy may arouse a concern of damage to the blood supply of the coracoid bone graft, and may result in graft absorption and nonunion [5].

Sufficient blood supply is an important factor for accelerating bone fracture healing [6]. Whether or not the traditional Latarjet osteotomy procedure causes damage to the blood supply of the CP is still controversial. The present study aims at providing the anatomical basis for the effect on blood supply of the CP as well as clarifying the effect of Latarjet osteotomy procedure on the blood supply of the coracoid bone graft by exploring the characteristics and changes in the blood supply before and after osteotomy.

## Materials and methods

### Subjects

The study was approved by the Ethics Committee at the First Hospital Affiliated to Shenzhen University, Shenzhen Second People’s Hospital, and was conducted in accordance with the World Medical Association Declaration of Helsinki. Five embalmed cadaveric upper limbs specimens with an average age of 42.6 years (ranging from 37 to 50 years) and 16 fresh frozen upper limbs with an average age of 43.7 years (ranging from 36 to 55 years) were purchased from the Department of Anatomy, Shenzhen University Medical School. The donors were free from shoulder diseases, congenital abnormality or injuries that harmed the normal structure and function of the shoulder. In order to reduce muscle and vessel damage, the upper limb samples were dissected from the trunk at the conjunction of ribs and spine so that the ribs could be kept attached to the shoulder.

### Embalmed cadaveric upper limb samples preparation

For the embalmed samples, the subclavian artery was perfused with red latex and then injected with 2 ml acetic acid for fixation. All the perforator arteries that related to the blood supply of the CP were carefully dissected for observation of their origins and courses. Samples of tendon–bone interface of CP and CT part (approximately 15 mm in length) were dissected, and then fixed in 10% neutral buffered formalin for 48 h. It was then decalcified with 7% hydrochloric acid for 48 h, alcohol dehydrated, and embedded in paraffin. Sections, 5-μm-thick, were cut in the sagittal view and stained with Hematoxylin and Eosin (HE). Deparaffinized slices were dyed with Hematoxylin for 5 min, after that 1-min water soak was given, and differentiated with 1% hydrochloric acid ethanol for 30 s, then a 15-min water soak was given and then the slices were dyed with 0.5% Eosin for 3 min, given a distilled water soak, and finally sealed for observation after dehydration. Finally, the specimens were observed for nutrient vessels distributions at the tendon–bone interface via light microscopy.

### Fresh frozen upper limb samples preparation

Sixteen fresh frozen upper limb samples were completely thawed in a 37°C water bath for 4 h, and then randomly divided into two groups for perfusion: the Latarjet osteotomy group (*n*=8), which underwent Latarjet osteotomy and the control group (*n*=8), which were kept intact.

The contrast agent preparation process was similar to a previous study [7]. Briefly, 30% barium sulfate and 5% gelatin suspension mixture was used as contrast agent. This agent was prepared by adding 40 g gelatin (V900863, Sigma, U.S.A.) into 800 ml water kept in an 80°C water bath. After it melted, the temperature of the water bath was decreased to 37°C and the gelatin suspension was added with 240 g barium sulfate (Blanc Fixe Micro, Sachtleben Chemie GmbH, mean grain size: 700 nm). The suspension turned to milky white color after sufficient stirring.

### Latarjet procedure and perfusion

For the intact group, a customized designed catheter (φ = 3 mm) was inserted into the exposed subclavian artery. A rubber band was tied strongly at the lower third of the upper arm to prevent the contrast agent from leaking. In order to infuse all the arteries in the samples, the pressure was maintained between 100 and 110 mmHg for at least 15 min during the perfusion, and all the leaking arteries were blocked by ligation or thermocoagulation. After that, the samples were kept in 0°C water bath for 4 h until the liquid contrast agent turned into gel state.

For the Latarjet osteotomy group, the harvest technique is based on Johanna’s technique [8]. Briefly, an incision of 25 mm was created at the tip of the CP in the downward direction. After blunt separation of the deltoid and pectoralis major, the tip of the CP was easily exposed at the insertion of the coracoclavicular (CC) ligaments at its base. The cephalic vein was carefully protected during the process. The CAL was identified when the shoulder was in abduction and external rotation position. The CAL was transected from its insertion on the CP. The CHL was also released. Subsequently, the position of shoulder was changed to adduction and internal rotation, where the PM tendon was released from its insertion on the medial aspect of the CP. Then a 90° oscillating saw was used to harvest the coracoid bone graft (18–22 mm from the coracoid tip) by performing a medial-to-lateral osteotomy at a position anterior to the insertion of the CC ligaments at the coracoid base. Ligation was performed in all the vessels during the process. Then the specimens were perfused and processed using the same method in the intact group.

### Coracoid bone graft samples acquisition

After the perfusion, CP in the intact group was exposed through a small vertical incision below the clavicle. CAL, CHL, PM and CT were exposed and cut off, leaving 5–10 mm of these structures attached on CP. The soft tissue attached on the superior and inferior side of the CP was preserved to check if it contained any vessels that supplied the CP. The procedure was carefully performed and all the white vessels were ligated with 3-0 suture to prevent it from leaking. Finally the CP was dissected at the conjunction of CP and scapula, then the whole CP was acquired for micro CT scanning.

**Figure 1:**
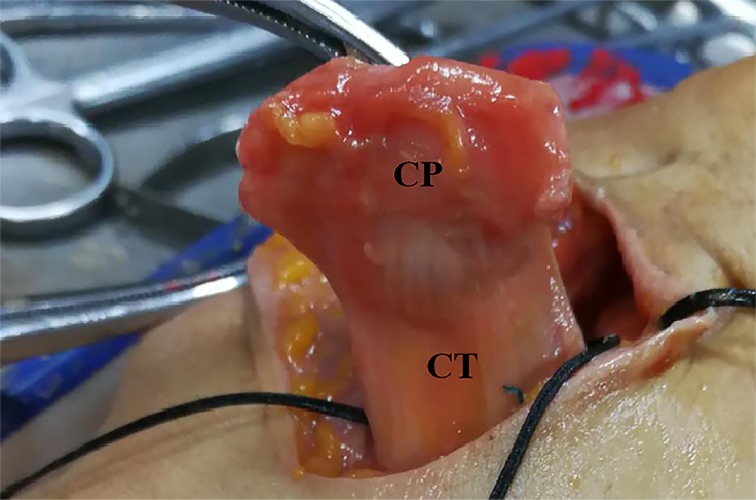
The free bone side of the CP and CT after Latarjet osteotomy procedure

For the Latarjet osteotomy group, the coracoid bone graft was acquired after dissection of the CT at the site of 3 mm length of the end attached to the CP (Figure 1).

### Micro CT evaluation

All the coracoid bone graft samples were scanned with a SkyScan 1176 Scanning System (Nanovea, U.S.A.) at 80 kV/300 μA and the resolution was 18 μm. The digitalized data were then imported into NRecon (Version1.6.10.1, offered by Bruker) to reconstruct images which would be imported into CTVox (offered by Bruker) for 3D reconstruction. Then 3D images of artery and bone were reconstructed and matched with respective artery distribution observed in embalmed specimens automatically. Changes in angiographic arteries between the intact group and osteotomy group were recorded.

## Results

### Macro observations of blood supply of the CP

**Figure 2:**
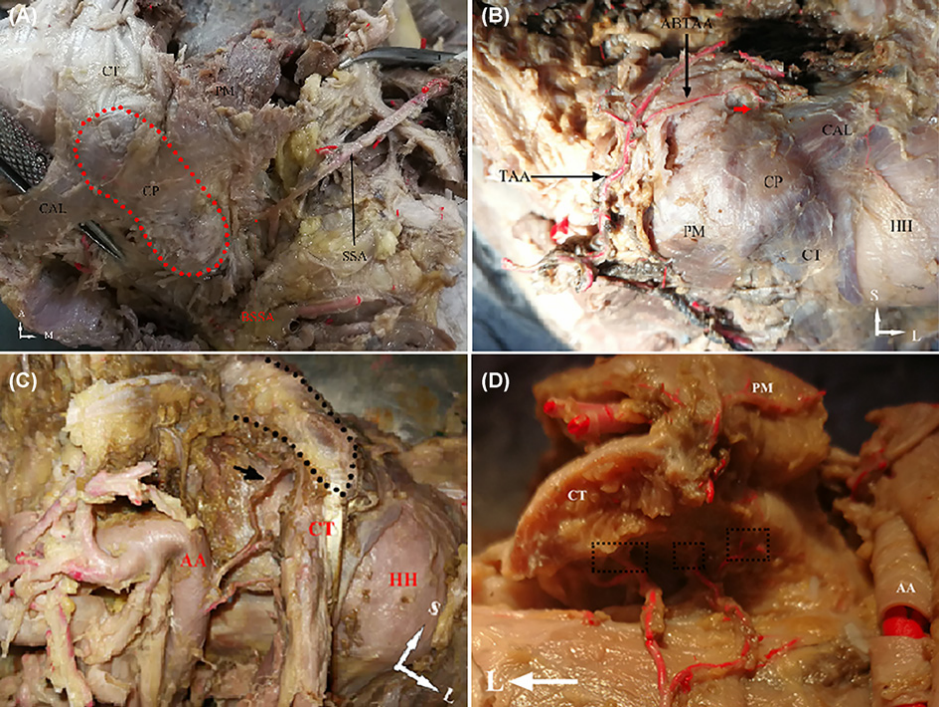
Macro observation of arterial supply of CP (**A**) Superior aspect of CP and SSA (left shoulder). (**B**) Superior aspect of antero-superior part of CP and its adjacent structures (left shoulder), the red thick arrow shows the branches of ABTAA. (**C**) Internal aspect of CP and CT with PM removed (left shoulder), the black thick arrow points to the branch that supplies the inferior side and the black dot outlines the CP. (**D**) Inferior aspect of CP with CT removed (right shoulder). The black dotted squares show the vessels that supply the inferior side. Abbreviations: BSSA, branch of SSA; HH, humeral head.

After careful observation of the embalmed specimens, we found that the CP’s arterial supply included branch of supra-scapular artery (SSA), acromial branch of thoraco-acromial artery (ABTAA) and branch derived from the second portion of axillary artery (AA). The SSA was derived from subclavian artery or thyrocervical trunk in some cases. The SSA coursed below and behind the clavicle toward the coracoid notch, then divided into two branches before it reached superior transverse scapular ligament (STSL). The first one ran beneath the STSL, then coursed poster-inferiorly to supply the supraspinatus muscle and infraspinatus muscle and ended up anastomosing with the circumflex scapular artery on the posterior surface of scapular neck. The second branch of SSA ran toward the medial side of the posterior part of CP as an intra-osseous nutrient artery (Figure 2A).

The TAA was mostly derived from the first portion of AA. After a short pathway along the medial side of PM, TAA ran superolaterally and then separated into several branches: the pectoral branch, deltoid branch and ABTAA. Four specimens were observed that the ABTAA gave a small branch artery penetrating into the CP’s upper side (Figure 2B).

In two specimens, an inconsistent branch from the second portion of AA ran supero-laterally between the PM and SUBS and ended up on the medial side of the CP (Figure 2C). In other two specimens, this branch had a different course and ended up on the inferior side of the CP (Figure 2D).

### Micro observations of blood supply of the CP

**Figure 3:**
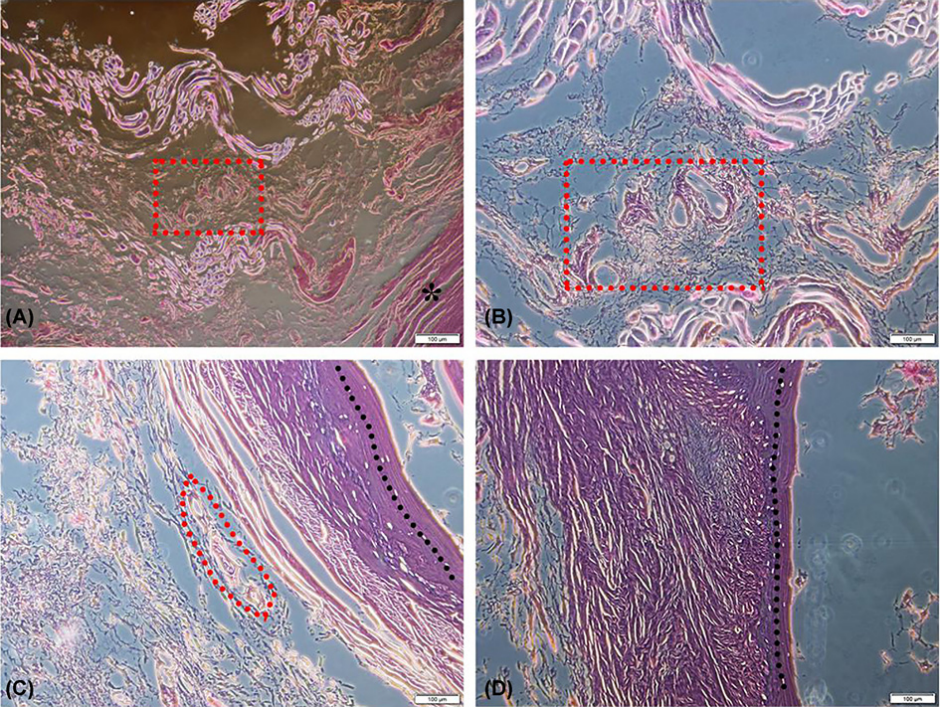
HE staining of the CP and CT interface in the embalmed cadaveric specimens (**A**) The red dotted square shows vessel distribution inside the CT. ‘*’ stands for cortical bone at the interface (40× field). (**B**) Enlarged image of vessel distribution inside the CT (100× field). (**C**) The red dotted area shows the nearest vessels close to the cortical bone after scanning all slides (100× field). (**D**) To the left of dotted line is the tendon-bone transition zone, and the cancellous bone is on the right side (100× field).

HE staining showed rich blood supply inside the CT, at the tendon–bone transition zone and CT interface in the embalmed cadaveric specimens. However, all the surface of the cortical bone in the interface was integrated, and no permeation of nutrient foramen blood vessel was found (Figure 3).

### Micro CT analysis

**Figure 4:**
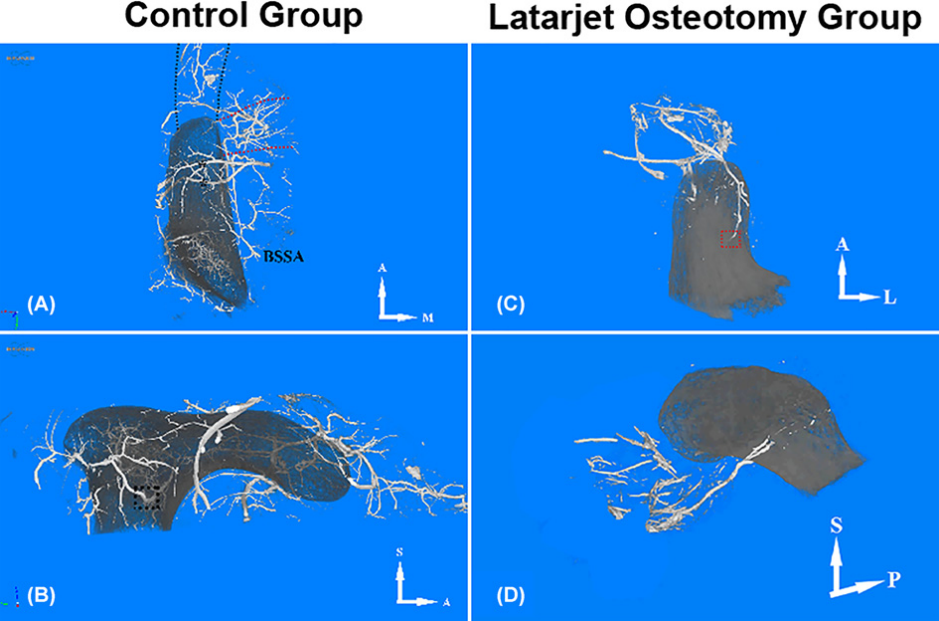
Micro CT 3D reconstruction of the vessel distribution of CP and its adjacent structures in the control group and the Latarjet osteotomy group On a right shoulder sample in the control group: (**A**) Rich vasoganglion is observed at the corresponding spatial position of CT and PM, but none of these vessels extended to the apex of CP. The black and red dotted line represents the CT and PM, respectively. (**B**) One sample showed a branch of an artery that penetrated the CP (black dotted square). However, this finding could not be seen on other samples in the control group. On a left shoulder sample in the Latarjet osteotomy group: (**C**). (**D**) Rich vasoganglion at the corresponding site of CT is seen, but only one vessel derived from CT penetrated the inferior side of CP. Abbreviations: A, anterior; BSSA, branch of SSA; L, lateral; M, medial; P, posterior; S, superior.

Precise images reflecting CP’s structure and its nutrient vasoganglion was presented in both groups after micro CT 3D reconstruction. On a right shoulder sample in the control group, we observed that the branches of SSA and branches of second portion of AA anastomosed and penetrated into the CP. However, no branch from ABTAA was found to supply the CP’s superior side in this specimen as we demonstrated in other embalmed specimens. No angiographic artery at the corresponding site of CAL or CHL was found in 3D images. Rich vasoganglion was observed at the corresponding spatial position of CT and PM, but none of these vessels extended to the apex of CP. The black and red dotted lines represented the CT and PM, respectively (Figure 4A). Interestingly, only one sample showed a branch of an artery that penetrated into the CP (black dotted square, Figure 4B). However, this finding could not be seen in other samples in the control group.

For the Latarjet osteotomy group, a left shoulder sample was presented. Although inferior quality of images were acquired due to bursting of the vessels during the perfusion, we could still observe rich vasoganglion at the corresponding site of the CT. Only in one sample it was found that one vessel derived from CT penetrated into the inferior side of CP (Figure 4C,D). This finding could not be seen in other samples, indicating that most of the blood supply into the coracoid bone graft was destroyed due to the Latarjet osteotomy procedure. As with the control group, these vasoganglion from the CT did not extend into the apex of CP.

## Discussion

**Figure 5:**
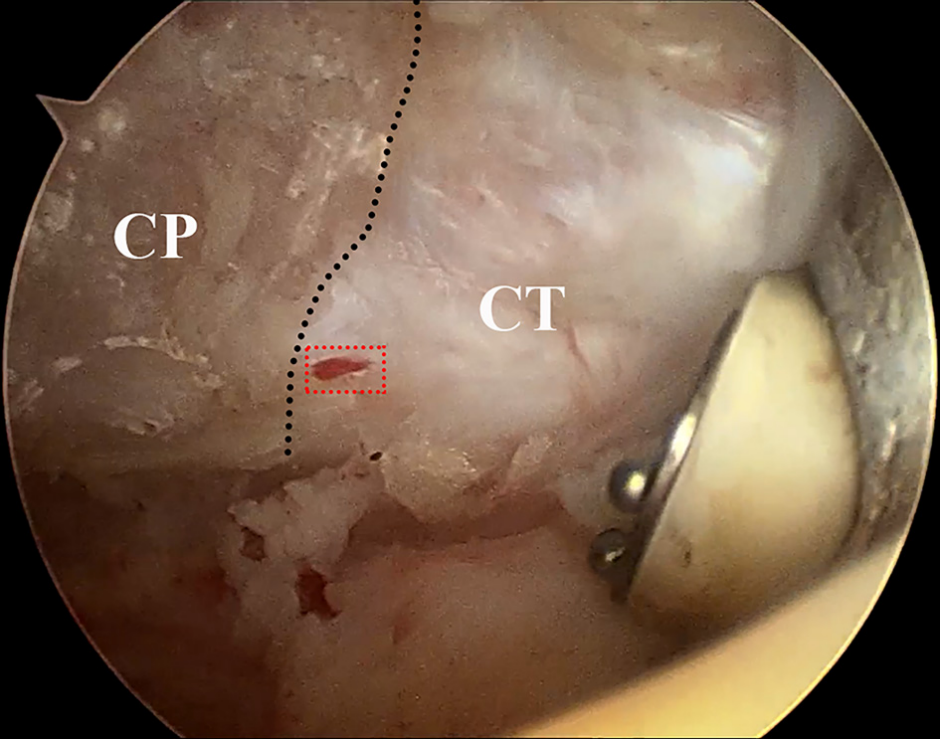
Rich vessels inside the CT near its attachment were observed under arthroscopy

In the present study, we found that the branch of SSA and the branch of ABTAA were relatively consistent nutrient arteries of the CP. Branches derived from the second portion of AA penetrated the CP on the medial side and supplied its horizontal part in some cases, while others had a variant of this branch which coursed on the inferior side of CP. No obvious sign of angiographic arteries was detected at the attachments of the CP, such as CAL and CHL. Conversely, angiographic arteries were observed from CT and PM, and contributed to the blood supply of the CP. Clinically, we can also recognize rich vessels inside the CT near its attachment under arthroscopy (Figure 5). For the Latarjet osteotomy group, one of the coracoid bone grafts showed that the blood vessels from the CT penetrated into the bone at the inferior wall. However, there was no successful angiographic artery penetrating the apex of the CP (attachment site of the CT). Therefore, it was believed that the CP owned a rich source of blood supply. Although inconsistent arteries derived from the CT could be seen, most of the blood supply of the coracoid bone graft was destroyed after osteotomy and transposition during the Latarjet procedure. This arouses the concern whether or not the blood supply of coracoid bone graft is preserved during the surgery.

Few studies have reported the blood supply of the CP before. According to Abrassart et al. [9], the blood supply of CP was partly provided by the branch of the SSA. A later study revealed the vascularization of the horizontal part of CP. Hamel et al. [10]found that the CP was nourished by the ABTAA, branch of the SSA, and inconsistently by a branch from the second portion of the AA, which penetrated the CP on the medial side and named this vessel ‘PM artery’. Our study resonates the same findings as Hamet et al. [10]. Furthermore, we found that rich vasoganglion inside the CT both before and after the Latarjet osteotomy procedure. Interestingly, we also observed a vessel that originated from the CT and penetrated the inferior wall of CP and nourished the coracoid bone graft after the osteotomy. However, this finding was only seen in one case, indicating that this could be an inconsistent branch arising from the second portion of AA that nourished the CP. Coincidentally, this artery once was recorded by Abrassart et al. [9] who named it the ascending glenoid artery (AGA). They reported that the AGA nourished SUBS, antero-superior capsule, glenohumeral ligament, CP and many other structures [9]. In our perspective, the direction of this vessel varied in individuals and possibly nourished PM, subscapularis muscle, glenohumeral ligament, CP and other structures.

Neither the open surgery nor the arthroscopic Latarjet procedure have changed the methods of harvesting the CP due to the surgery’s principle [11,12]. It is important to preserve the blood supply of coracoid bone graft for reducing complications such as bone absorption and bone nonunion. It was impossible to preserve branches of ABTAA and branches from second portion of AA because of its short course and the thickness of subscapularis during osteotomy. Only vessels that originated from the CT could possibly be retained. During the traditional graft transposition and fixation technique, the inferior wall of the coracoid bone grafts was decorticated and fixed on the glenoid, which is bound to destroy this nutrient artery. During the congruent-arc transposition and fixation technique, the coracoid bone graft was transferred and fixed on the glenoid after internal rotation of 90°. This made the inferior wall to be a part of new joint surface [13]. Therefore, it is only possible to preserve the arteries that penetrated the inferior wall of CP by congruent-arc technique combined with prohibition of early active movement of abduction and external rotation to reduce the impact of humeral head on the graft. When these two techniques were combined desired clinical results were achieved [14,15]. However, due to the inconsistent occurrence of these vessels, it is difficult to identify the role of inferior penetrating artery on reducing the incidence of osteolysis and nonunion.

The existence of blood supply from CT to CP was a topic of controversy for a long period. In an observational study, Zhu et al. [16]speculated that the coracoid bone graft may get blood supply from the CT and soft tissue attached on it after Latarjet procedure was performed, while more convinced evidence was lacking. Conversely, Hamel et al. [17] did not find such vessels coming from the CT to supply the CP in a cadaveric study. To the best of our knowledge, the present study is first of its kind to provide sufficient evidence and prove the presence of nutrient vessels originating from CT to CP post-osteotomy by angiographic study.

After osteotomy, the traditionally fixed bone graft fixed is still without blood supply. Our findings may change the clinical perspective of some sports medicine doctors, such as the choice of Latarjet osteotomy or iliac crest bone graft transfer (ICBGT). The Latarjet osteotomy is considered superior to ICBGT in some degree because of the blood supply from CT. Theoretically, the rate of nonunion and bone resorption after Latarjet procedure should be lower than that of ICBGT. However, Moroder et al. [18]reported that there was no significant differences in clinical and radiological outcomes between the two procedures, indicating whether blood supply from CT exists or not was not the main reason for bone healing and absorption. Interestingly, in cases of application of ICBGT as bone graft, Ettore et al reported a modified fixation method using button achieved satisfactory outcome and higher bone healing rate than that using screw fixation [19,20]. Thus, we propose to shift the future research focus from bone grafts selection to fixation methods selection.

Limitations of the present study should also be acknowledged. Firstly, the sample size is relatively small. In order to draw more definitive conclusions, larger scale investigations need to be carried out. Second, the effect of perfusion was not perfect and many small vessels might be detected if advanced perfusion and radiology techniques were applied, which would be the future directions of our study.

In conclusion, the CP is a structure with rich blood supply, and is nourished by branches of TAA, and the second portion of AA consistently. The traditional Latarjet procedure would inevitably destroy the blood supply of the coracoid bone graft.

## Availability of Data and Materials

All data generated or analyzed during the present study are included in this published article.

## Abbreviations

AA: axillary artery
ABTAA: acromial branch of thoraco-acromial artery
AGA: ascending glenoid artery
CAL: coracoacromial ligament
CC: coracoclavicular
CHL: coracohumeral ligament
CP: coracoid process
CT: conjoint tendon
ICBGT: iliac crest bone graft transfer
PM: pectoralis minor
SSA: supra-scapular artery
STSL: superior transverse scapular ligament
SUBS: subscapular muscle
TAA: thoraco-acromial artery
